# UBE2C is a diagnosis and therapeutic biomarker involved in immune infiltration of cancers including lung adenocarcinoma

**DOI:** 10.7150/jca.92473

**Published:** 2024-01-27

**Authors:** Daxia Cai, Feng Tian, Minhua Wu, Jianfei Tu, Yonghui Wang

**Affiliations:** 1 Cancer Center, Lishui Central Hospital, The Fifth Affiliated Hospital of Wenzhou Medical University, Lishui Hospital of Zhejiang University, Lishui 323000, China.; 2 Department of Stomach Enterochirurgia, Lishui People's Hospital, the Six Affiliated Hospital of Wenzhou Medical University, Lishui, Zhejiang, China.

**Keywords:** UBE2C (Ubiquitin-conjugating enzyme E2C), LUAD (Lung adenocarcinoma), immune infiltration, biomarker, diagnosis

## Abstract

The mechanism of action of UBE2C in lung adenocarcinoma (LUAD) and its significance in cancer diagnosis, targeted therapy and immunotherapy, even in pan-cancer, are still unclear. Several large public databases and online analysis tools were used for big data mining analysis. RNA interference technology, CCK8 assay, flow cytometry and apoptosis detection, and western blot were used for in vitro experiments. UBE2C was found to be overexpressed in various of tumors, including LUAD, and its expression level was found to be significantly related to gender, weight, tumor stage, grade and prognosis in LUAD. Downregulation of UBE2C expression induced proliferation suppression and G2/M phase arrest and cell apoptosis in LUAD cells and suppressed LUAD cell growth through inhibiting the Akt-mTOR signaling pathway. Expression level of UBE2C was negatively correlated with B cells and CD4+ T cell, and also with immune checkpoint genes in LUAD. Pan-cancer assay shown that UBE2C was significantly overexpressed in 28 cancers and was correlated with Ki-67 index in many cancers. Overexpression of UBE2C in BRCA, LUAD and MESO indicated worse Overall Survival (OS). UBE2C expression levels were positively associated with immunocyte infiltration, immune regulatory genes, immune checkpoints, TMB, MSI and MMRs in some cancers. Additionally, Single-cell functional analysis showed that UBE2C was positively correlated with cell cycle, proliferation, DNA damage, EMT, DNA repair, invasion and differentiation in some cancers. These findings suggested that UBE2C could be regarded as a latent diagnosis and prognostic biomarker and a new target for immunological therapy of cancers including LUAD.

## Introduction

Lung adenocarcinoma (LUAD), as a leading causes of cancer-related death worldwide, has attracted significant attention. Despite the significant advancements in the diagnosis and treatment of LUAD, the five-year survival rate remains quite low due to challenges such as late detection, high rate of metastasis, drug resistance, and the absence of systematic treatment 1. Therefore, it's crucial to comprehend the molecular mechanisms underlying its occurrence and progression, seek out new molecular markers, and enhance the diagnostic and therapeutic levels of LUAD.

Ubiquitin-conjugating enzyme E2C (UBE2C, also known as UBCH10) is known to be an essential part of the ubiquitin proteasome system, constituting a conserved core domain containing the catalytic Cys residue and an N-terminal extension 2. Periodically activated during the cell cycle, initiates the ubiquitination of cell cycle-related proteins and is required for the destruction of mitotic cyclins and safety proteins necessary for spindle assembly checkpoints and mitotic exit, thereby playing a role in promoting cell mitosis 3, 4. Studies have shown that UBE2C promotes proliferation and progression of breast cancer through AKT/mTOR signaling pathway 5. However, the mechanism of action of UBE2C in LUAD and its implications in diagnosis, targeted therapy of LUAD and immunotherapy, and even in pan-cancer, remain unclear. Therefore, this study aims to elucidate the potential mechanism by which UBE2C affects the proliferation of LUAD and even pan-carcinoma, as well as its crucial role in cancer diagnosis, targeted therapy and immunotherapy.

In this study, we have demonstrated that overexpression of UBE2C promotes the proliferation, metastasis, and invasion of LUAD cells. Decreasing UBE2C expression inhibited cell proliferation, induced G2/M phase arrest, and triggered cell apoptosis in LUAD cells. Furthermore, it suppressed LUAD cell growth by inhibiting the Akt-mTOR signaling pathway.

Additionally, UBE2C expression can serve as a predictive marker for the effectiveness of immune checkpoint inhibitors in LUAD patients. Immune checkpoint inhibitors include anti-CD274, anti-CTLA4, anti-HAVCR2, anti-LAG3, anti-PDCD1, anti-PDCD1LG2, and anti-TIGIT. Moreover, we conducted a comprehensive investigation into the association between UBE2C and transcriptional level, clinical prognosis, immune infiltration, tumor microenvironment (TME), tumor mutation load (TMB), immune infiltration, microsatellite instability (MSI), mismatch repair (MMR) in 33 types of cancer. This study provides valuable insights and directions for future research on UBE2C -based anti-tumor therapy research. Overall, our study validated UBE2C as a promising biomarker for cancer diagnosis and prognosis, as well as a target for cancer therapy.

## Materials and Methods

### Databases and online analysis tools

The expression microarray series of UBE2C encoding LUAD tumor and non-tumor samples were downloaded from the Gene Expression Omnibus (GEO, https://www.ncbi.nlm.nih.gov/geo/) database. These included: GSE1037 (GPL962) 6, GSE2088 (GPL962) 7, GSE7670(GPL96), GSE10072 (GPL96) 8, GSE19188(GPL570) 9, GSE31210 (GPL570) 10, GSE31908 (GPL96), GSE32863 (GPL6884) 11, GSE40275 (GPL15974) 12, and GSE116959 (GPL17077) 13.

The GEPIA2 14 (Gene Expression Profiling Interactive Analysis) online database (http://gepia2.cancer-pku.cn/#index) is used for UBE2C differential expression analysis between LUAD and normal samples. The Kaplan Meier plotter 15 is capable of assessing the correlation between the expression of UBE2C and survival. LinkedOmics 16 (http://www.linkedomics.org) is a publicly available portal for clinicians and biologists to analyze and compare cancer multi-omics data. TIMER 17 (Tumor Immune Estimation Resource) (https://cistrome.shinyapps.io/timer/) is for comprehensive analysis of tumor-infiltrating immune cells across diverse cancer types. The expression of UBE2C in common human LUAD cell lines were downloaded from the Cancer Cell Line Encyclopedia (CCLE) database (https://sites.broadinstitute.org/ccle). UALCAN 18 is used to analyze UBE2C expression based on total protein. Metascape 19 (http://metascape.org) is used for enrichment analysis. Human Protein Atlas (https://www.proteinatlas.org/) is an open access resource for human proteins. Cancer single-cell state Atlas (CancerSEA, http://biocc.hrbmu.edu.cn/CancerSEA) is for studying cancer cell functions at the single-cell level 20.

### Cell cultures and transfections

The LUAD cell lines NCI-H1299 and A549 were purchased from the American Type Culture Collection (ATCC; Manassas, VA, USA), for their established relationships with LUAD, a type of lung cancer. These cells were then cultured in Dulbecco's modified Eagle's medium (DMEM) supplemented with 100 µg/mL streptomycin (HyClone, Logan, UT, USA), 100 U/mL penicillin (HyClone) with d 10% fetal bovine serum (FBS; Gibco, Life Technologies, Carlsbad, CA, USA) in a humidified incubator at 37 ◦C with 5% CO2.

To knock out UBE2C in these cell lines, small interfering RNA (siRNA) (Genepharma, Shanghai, China) was used. The specific target sequences for UBE2C of A549 cells were siUBE2C (5′-CUUCUAGGAGAACCCAACA-3′), and for NCI-H1299 cells were siUBE2C (5'-GTTCCTCACGCCCTGCTAT-3'). These cells were then selected for transfection, and Lipofectamine™ 3000 Transfection Reagent (Thermo Fisher Scientific, Waltham, MA, USA) was used in accordance with the manufacturer's instructions.

### Quantitative reverse transcription PCR (RT-qPCR) assay

The total RNA from A549 and NCI-H1299 cells was extracted after using TRIzol reagent, which was then used for reverse transcription to generate cDNA. This cDNA was then utilized in real-time quantitative polymerase chain reaction (RT-qPCR) experiment. In the PCR system, the following reagents were involved: 0.2μL of forward primer, 0.2μL of reverse primer, 2μL of cDNA, 10μL of SYBR Premix Ex TaqTM II (TaKaRa, Japan) and double distilled water (ddH2O) was added up to a final volume of 20μL. The first step is a denaturation at 95°C for 30 seconds, followed by PCR amplification at 95°C for 3 seconds, and then at 60°C for 30 seconds, totaling 40 cycles, after which we enter the separation phase. The internal control gene selected in this study was β-Actin. The sequences of the primers used to assess UBE2C expression were as follows: UBE2C forward primer (5′-CTGCCTTCCCTGAATCAGACAACC-3′), UBE2C reverse primer (5′-TCGGCAGCATGTGTGTTCAAGG-3′), β-Actin forward primer (5′-CTCCATCCTGGCCTCGCTGT-3′), and β-Actin reverse primer (5′-GCTGTCACCTTCACCGTTCC-3′).

### Western blotting analysis, Cell proliferation assay and cell cycle/apoptosis assay

The Western blotting procedure was conducted as described previously 21. Antibodies used in our study included anti-UBE2C (1:2000; ab125002), anti-human β-actin (1:1000, #4970; Cell Signaling Technology, Danvers, MA, USA), anti-p-AKT (1:1000; CST4060), anti-AKT (1:1000; CST4691), anti-p-mTOR (1:1000; CST5536), anti-mTOR (1:1000; CST2972), CDK1/2 (AN21.2, Santa Cruz Biotechnology, sc-53219), PCNA (PC10, Santa Cruz Biotechnology, sc-56), MCM2 (E-8, Santa Cruz Biotechnology, sc-373702), MCM4(G-7, Santa Cruz Biotechnology, sc-28317), MCM6 (H-8, Santa Cruz Biotechnology, sc-393618), MCM7 (141.2, Santa Cruz Biotechnology, sc-9966), CASP3(caspase-3, 31A1067, Santa Cruz Biotechnology, sc-56053), DFFA (LCAD, F-8, Santa Cruz Biotechnology, sc-17816), MAP2K2 (A-1, Santa Cruz Biotechnology, sc-13159), LMNB2 (F-8, Santa Cruz Biotechnology, sc-377379), TRADD (A-5, Santa Cruz Biotechnology, sc-46653) and anti-rabbit IgG (1:1000; #7074, Cell Signaling Technology).

Cell proliferation assay was conducted employing the Cell Counting Kit 8 (CCK-8) assay, purchased from Beijing Solar Science & Technology Co., Ltd., Beijing, China. Additionally, an established protocol for cell cycle/apoptosis analysis was followed, as previously described 21, A CS012(Multisciences (Lianke) Biotech Co., Ltd., Hangzhou, China) cell cycle staining solution and AP101(Multisciences (Lianke) Biotech Co., Ltd.) cell apoptosis staining solution were then applied. Flow cytometry was used to evaluate the cell suspension, employing the ACEA NovoCyte flow cytometer and NovExpress software (Agilent Technologies, Santa Clara, CA, USA). These experiments were all conducted in triplicates.

### Statistical methods

Student's t-test or Wilcoxon rank-sum test were used to evaluate all continuous variables. Statistical analysis and visualization were conducted using GraphPad Prism software version 8.0 (GraphPad Software, Inc., San Diego, CA, USA. A significance level of p < 0.05 was considered statistically significant.

## Results

### UBE2C was significantly overexpressed in LUAD

**Figure 1:**
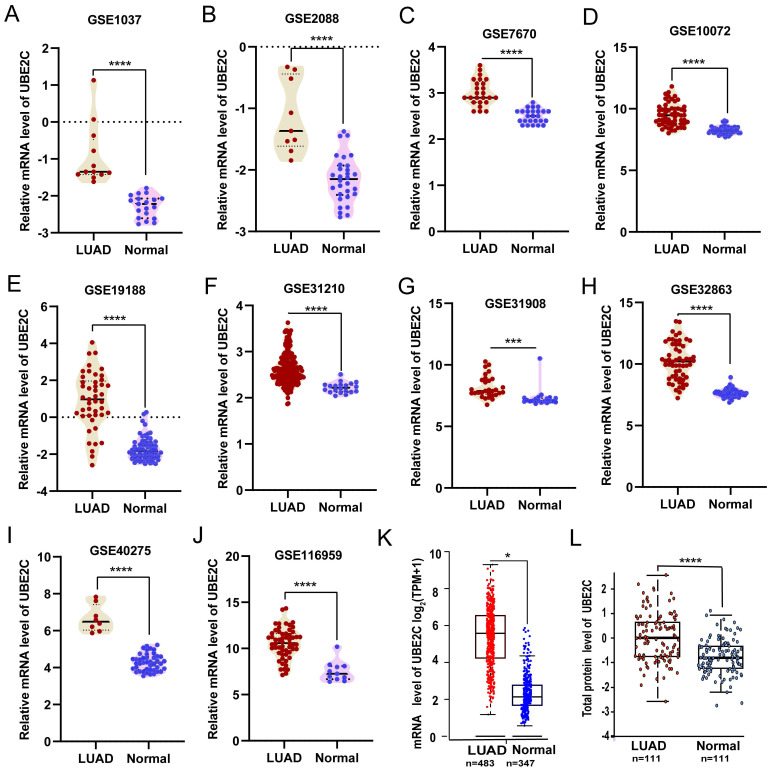
The different expression of UBE2C in LUAD. (A-J) Comparisons of UBE2C mRNA expression between normal and LUAD tissues were conducted using data obtained from the GEO database (Student's t-test). (K) UBE2C mRNA expression was compared between normal and LUAD tissues in TCGA-LUAD cohort using GEPIA2 online database (unpaired Wilcoxon test). (L) Protein expression of UBE2C was compared between normal and tumor tissues using the UALCAN web tool (Wilcoxon test). UBE2C = Ubiquitin conjugating enzyme E2 C; LUAD = lung adenocarcinoma. TCGA = The Cancer Genome Atlas; *p*-value < 0.05 was used to assess differences.

Compared with the matched normal tissues, UBE2C was significantly overexpressed in LUAD from ten GEO validation sets (Figures 1A-J; *p*<0.001) and GEPIA2 database (Figure 1K; *p*<0.05) based on mRNA expression. Then, we confirmed the above conclusions from the protein expression level through the UALCAN database (Figure 1L; *p*<0.001).

### Distribution of UBE2C expression in clinical characteristics sub-groups of LUAD

**Figure 2:**
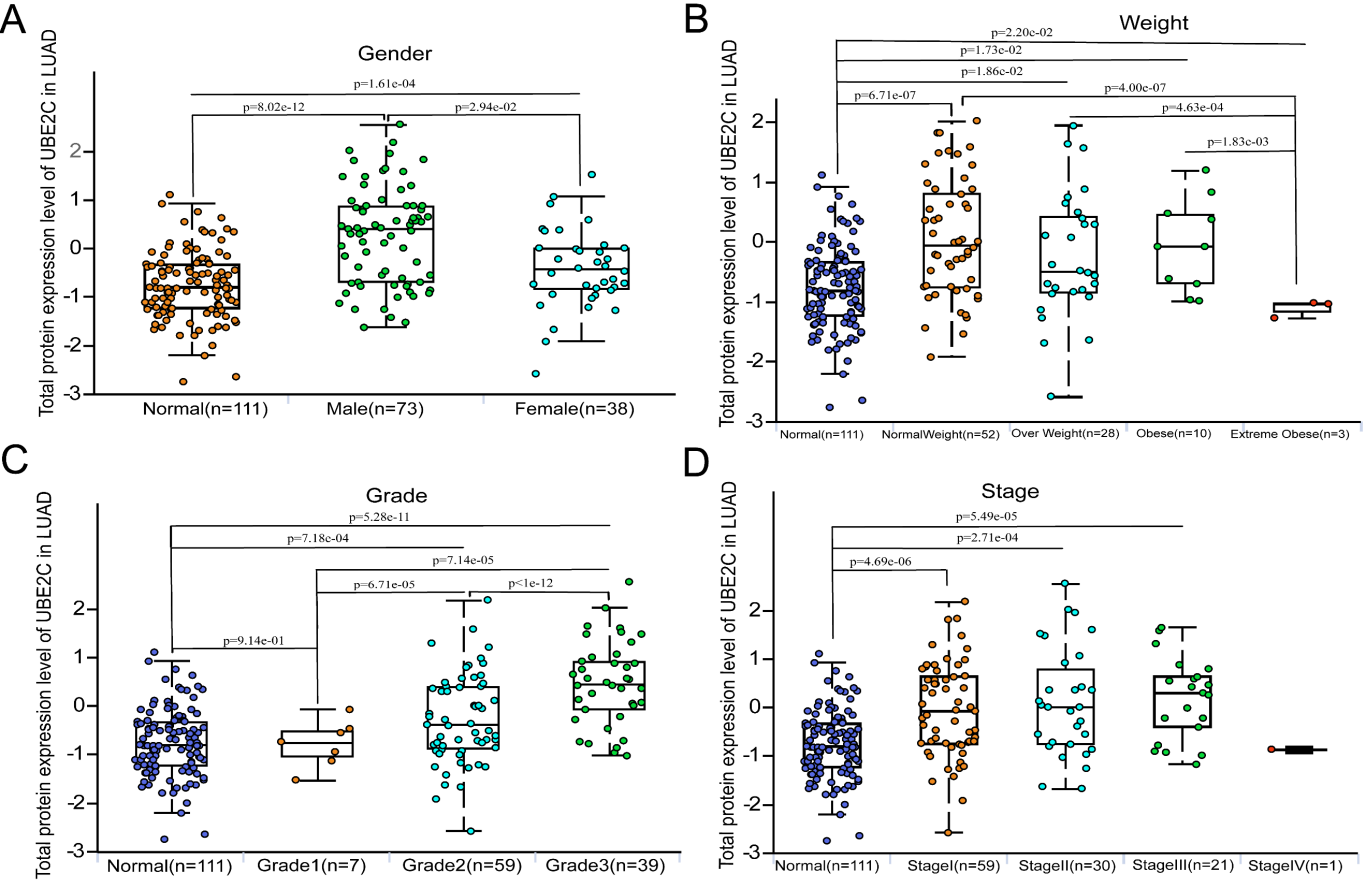
Distribution of protein expression of UBE2C in clinical characteristics sub-groups of LUAD. (A) Gender. (B) Weights. (C) Grade. (D) Stage. UBE2C = Ubiquitin conjugating enzyme E2 C.

We observed that the expression of UBE2C in male patients with LUAD was significantly higher than that in female patients (p = 2.94e-02, Figure 2A). Furthermore, significant differences in UBE2C expression were found among LUAD patients with different body weights (Figure 2B). Specifically, the expression of UBE2C was significantly lower in the Extreme Obesity group compared to the Normal Weight group (p=4.0e-07), Overweight group (p =4.63e-04), and Obese group (p = 1.83e-03). Additionally, we found that higher tumor grades in LUAD patients correlated with higher UBE2C expression (Figure 2C;* p*<0.001). Moreover, UBE2C expression was observed to increase with the advance of the disease stage (Figure 2D). Due to the limited number of Stage IV LUAD patients, further analysis was not conducted on this subgroup. Collectively, these findings suggest that UBE2C overexpression may contribute to the metastasis and invasion of LUAD. Additionally, significant differences in UBE2C expression were detected among LUAD patients based on gender and weight (Figure 2).

### High expression of UBE2C is a potential independent risk factor in LUAD

In a multivariate Cox-regression analysis conducted to explore the relationships between clinicopathological factors and OS in LUAD patients, we found that UBE2C overexpression, gender, advanced American Joint Committee on Cancer (AJCC) stage, grade and smoking history should be potential risk factors for OS (all *p* < 0.05). UBE2C overexpression group had a significantly higher proportion of male cases (*p* = 3.6e-10, Table 1).

Additionally, we conducted a gender-based comparison of UBE2C total protein expression levels. Our results indicated that UBE2C protein was found to be significantly overexpressed in male compared to female (*p* = 2.94e-02, Figure 2A). Furthermore, when comparing patients with AJCC stage I, those with AJCC stage II and stage III had significantly higher UBE2C total protein expression levels (Figure 2D). What's more, both Grade3 and Grade2 exhibited significantly higher UBE2C total protein expression levels than Grade1(*p* =7.14e-05 and *p* = 6.71e-05, respectively, Figure 2C). Additionally, Grade3 showed significantly higher UBE2C total protein expression levels than Grade2 (*p*<1e-12, Figure 2C).

All above results indicate that high expression of UBE2C may serve as a potential independent risk factor for patients with LUAD.

### High expression of UBE2C is a potential biomarker for the diagnosis and prognosis of LUAD

**Figure 3:**
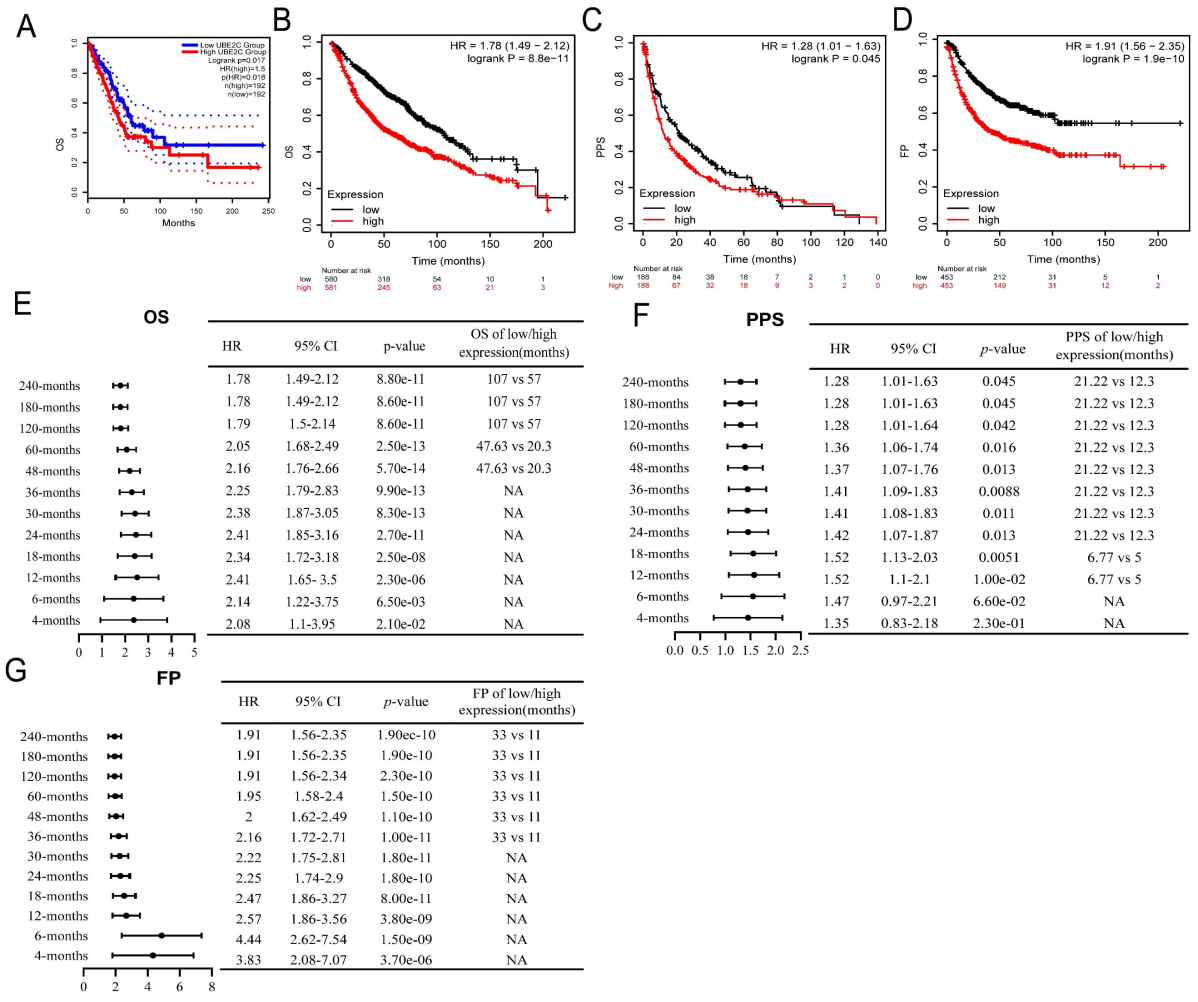
Relationship between UBE2C expression and prognosis of LUAD patient. (A-B) Relationship between UBE2C expression and OS in LUAD patients through GEPIA2 database (A) and Kaplan Meier plotter (B). (C-D) Relationship between UBE2C expression and PPS (C) and FP(D) in LUAD patients through Kaplan Meier plotter. (E-G) Relationship between the expression of UBE2C and the OS(E), (PPS)(F) and FP(G) different clinical characteristics sub-groups of LUAD by forest map. OS: Overall Survival; FP: First Progression; PPS = Post Progression Survival. *p* < 0.05 was used to assess differences.

The overexpression of UBE2C in LUAD tissues was significantly associated with poor OS [HR=1.5, log rank p = 0.017, Figure 3A]. This finding was corroborated by the results of a Kaplan-Meier plotter online analysis tool, which also confirmed a similar HR of 1.5 and a 95% CI of 1.49-2.12 (log rank p = 8.8e-11, Figure 3B). Additionally, UBE2C overexpression was associated with poor Post Progression Survival (PPS) [HR = 1.28, 95%CI=1.01-1.63, log rank p = 0.045, Figure 3C] and first progression (FP) [HR = 1.91, 95%CI=1.56-2.35, log rank p = 1.9e-10, Figure 3D] in LUAD patients. Subgroup analyses furthermore indicated that UBE2C upregulation was a risk factor for 4-months, 6-months, 12-months, 18-months, 24-months, 30-months, 36-months, 48-months, 60-months, 120-months, 180-months, and 240-months OS (Figure 3E), PPS (Figure 3F), and FP (Figure 3E-G;* p*<0.05). All these results suggested that UBE2C overexpression predicts poor prognosis in LUAD patients, underscoring the importance of UBE2C in LUAD pathogenesis and suggesting potential therapeutic targets.

In an effort to further explore the significance of UBE2C in the diagnosis of LUAD, mRNA data of LUAD and normal lung tissues were downloaded from GEO database for ROC analysis. We found that expression of UBE2C exhibited relatively high diagnostic efficiency, with a high sensitivity, specificity and cut-off value (Supplementary Figure 1, all *p*<0.0001). This finding suggests that expression of UBE2C might be sensitive biomarker for diagnosis of LUAD and could potentially serve as precise therapeutic targets for LUAD.

### Co-expression and enrichment analysis of UBE2C in LUAD

**Figure 4:**
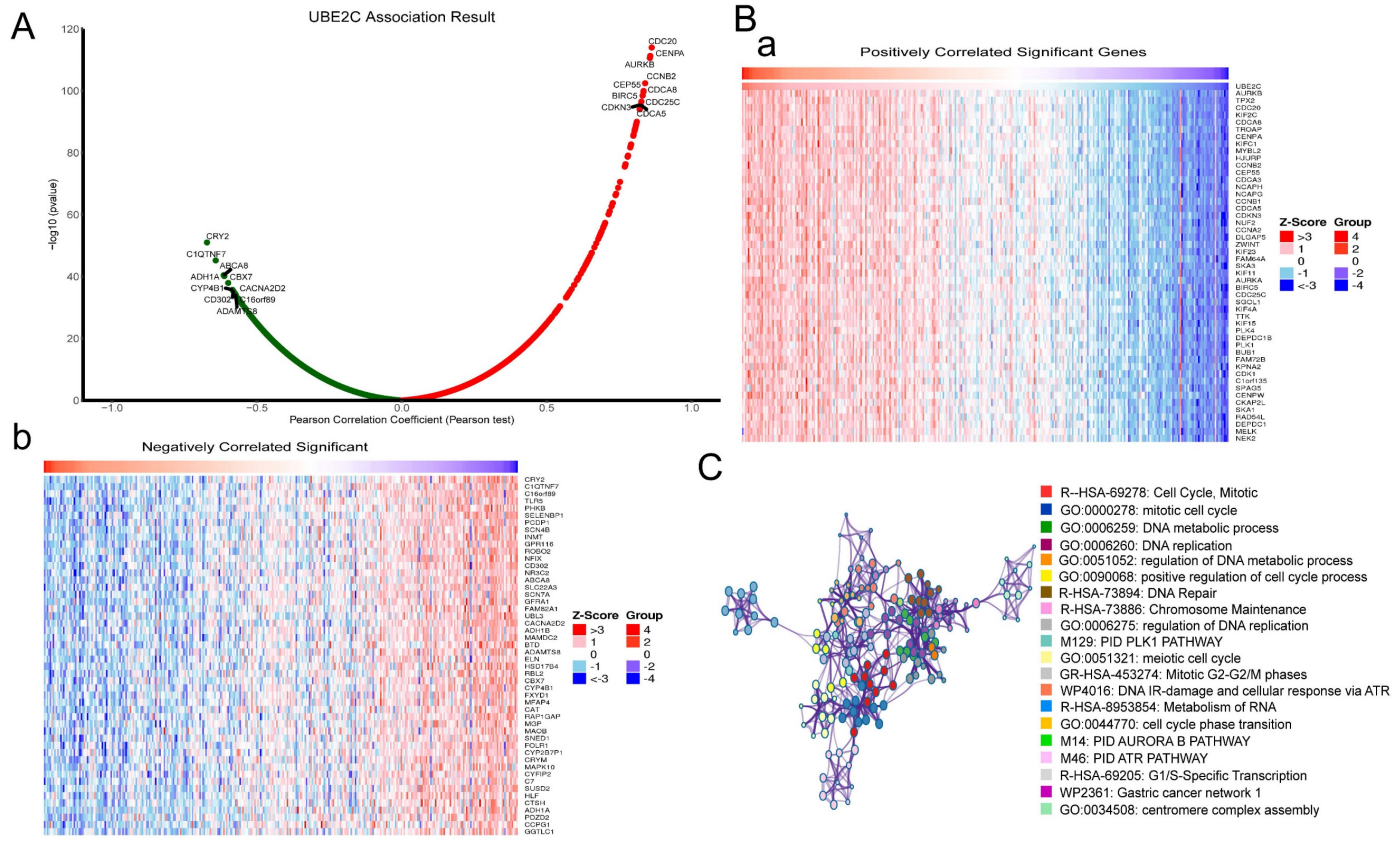
Functional enrichment analysis of UBE2C in the development of LUAD. (A) The global UBE2C highly co-expressed genes identified by the Spearman test in LUAD (LinkedOmics). Red and green dots represent positively and negatively significantly correlated genes with UBE2C, respectively. (B a-b) Heatmaps showing the top 50 genes positively and negatively correlated with UBE2C in LUAD (LinkedOmics). (C) Enrichment analysis of UBE2C co-expression genes in LUAD through Metascape database.

In order to understand the mechanism of UBE2C in LUAD, the LinkedOmics database was used to analyze UBE2C co-expression genes in LUAD. As plotted in Figure 4A, 19962 genes were correlated with UBE2C in LUAD (p-value<0.05). Heatmaps of the top 50 genes positively and negatively respectively associated with UBE2C as showed in Figure 4B(a-b). Then, Metascape database was used to explore the enrichment analysis of these genes, and the results showed that these genes were mainly enriched in Cell Cycle, Mitotic, DNA metabolic process, DNA replication, regulation of DNA replication, Mitotic G2-G2/M phases, and so on (Figure 4C). Therefore, it's believed that UBE2C may promote the progression of LUAD by regulating cell cycle, apoptosis and other mechanisms, thus affecting the prognosis of LUAD patients.

### Downregulation of UBE2C expression inhibited proliferation and induced G2/M phase arrest and cell apoptosis in LUAD cells

Previously, we predicted that UBE2C might promote LUAD development by participating in cell cycle progression and cell apoptosis. To confirm this hypothesis, we conducted in vitro experiments.

**Figure 5:**
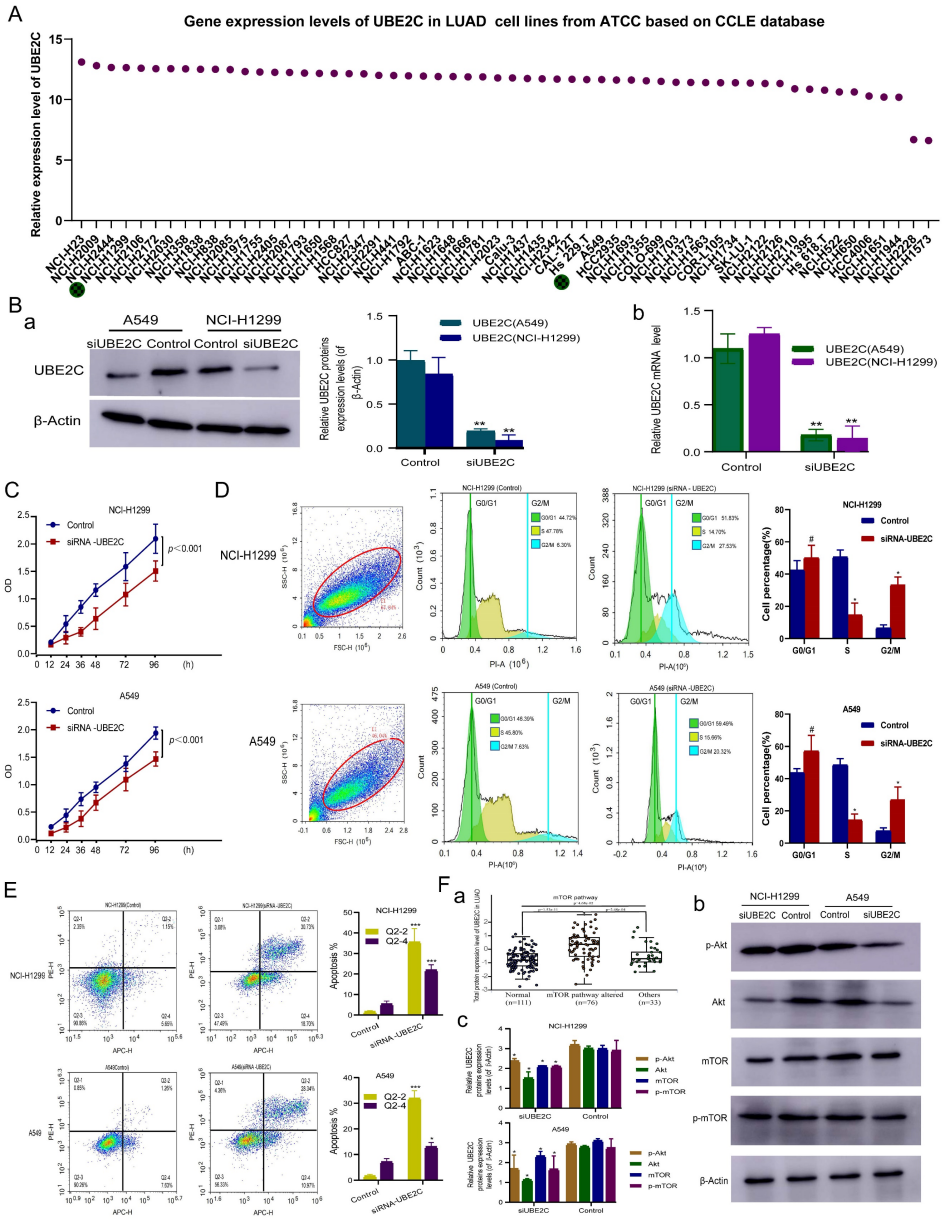
In vitro experiments to explore the molecular mechanism of UBE2C in LUAD. (A) Gene expression levels of UBE2C in common human LUAD cell lines from the Cancer Cell Line Encyclopedia (CCLE) database. (B) The protein and mRNA expression levels of UBE2C detected after siRNA transfection in A549 and NCI-H1299 cells. (C) Effect of UBE2C siRNA knockdown on the proliferation of NCI-H1299 and A549 cells by Cell Counting Kit 8 assay. (D-E) Effect of UBE2C siRNA knockdown on NCI-H1299 and A549 cell cycle (D) and cell apoptosis (E) by flow cytometry. (F) siUBE2C inhibits the PI3K-Akt-mTOR signaling pathway in LUAD: (a) UBE2C expression and somatic changes in Akt-mTOR signaling pathway using proteomics; (b) NCI-H1299 and A549 cells were transiently transfected with siUBE2C for 48 h, and the expression of Akt, phospho-Akt, mTOR and phospho-mTOR was then analyzed by Western blot. β-Actin was used as a loading control. LUAD = Lung adenocarcinoma (^*^p < 0.05, ^**^p < 0.01, ^***^p < 0.001, ^****^p < 0.0001).

In order to select suitable LUAD cell lines for construction of siRNA- UBE2C, we downloaded the expression level of UBE2C in common human LUAD cell lines from CCLE database from ATCC cell line bank for further analysis, and the results showed that except for NCI-H2228 and NCI-H1573, UBE2C was expressed at similar levels in other 56 LUAD cell lines (Figure 5A). Given the availability of cell lines, NCI-H1299 and A549 were selected as the experimental lines for this study.

To investigate the function of UBE2C in LUAD progression and development, siRNA (siUBE2C) knockdown of UBE2C in A549 and NCI-H1299 cells was performed. The results of Western-blotting [Figure 5B(a)] and RT-qPCR [Figure 5B(b)] assay revealed that the protein and mRNA expression levels of UBE2C were significantly reduced in both A549 and NCI-H1299 (*p* < 0.001, *p* < 0.001) UBE2C knockdown cells (Figure 5B).

NCI-H1299 and A549 cells were cultured after they were transfected with siRNA UBE2C, respectively. CCK-8 assay was used to detect the effect of UBE2C on the proliferation of LUAD cell lines (NCI-H1299 and A549), which showed that the activity of NCI-H1299 cells and A549 were significantly reduced from 12 to 96 hours after transfection with siUBE2C, respectively (Figure 5C; *p*<0.001).

Cell cycle and apoptosis of NCI-H1299 and A549 after UBE2C downregulation were detected by flow cytometry, respectively (Figure 5D-E). After transfection with siRNA -UBE2C, the proportion of NCI-H1299 cells in the G2/M phase increased from 6.30% to 27.53% (*p* < 0.05), while that of cells in the S phase concomitantly decreased from 47.78% to 14.70% (*p* < 0.05) and that of cells in the G0/G1 phase increased from 44.72% to 51.83% (*p*>0.05) (Figure 5D). Meanwhile, the proportion of A549 cells in the G2/M phase increased from 7.63% to 20.32% (*p* < 0.05), while that of cells in the S phase concomitantly decreased from 45.80 % to 15.66 % (*p* < 0.05) and that of cells in the G0/G1 phase increased from 46.39 % to 59.49 % (*p*>0.05) after transfection with siRNA-UBE2C (Figure 5D). The above results indicate that downregulation of UBE2C expression substantially induced G2/M phase arrest both in NCI-H1299 and A549 cells, respectively. Additionally, when NCI-H1299 and A549 cells were treated with siRNA - UBE2C, respectively, the proportion of early apoptosis and late apoptosis cells all significantly increased (*p* < 0.001,* p* < 0.05, respectively; Figure 5E). The results suggest that UBE2C effectively inhibited apoptosis in NCI-H1299 and A549 cells.

**Figure 6:**
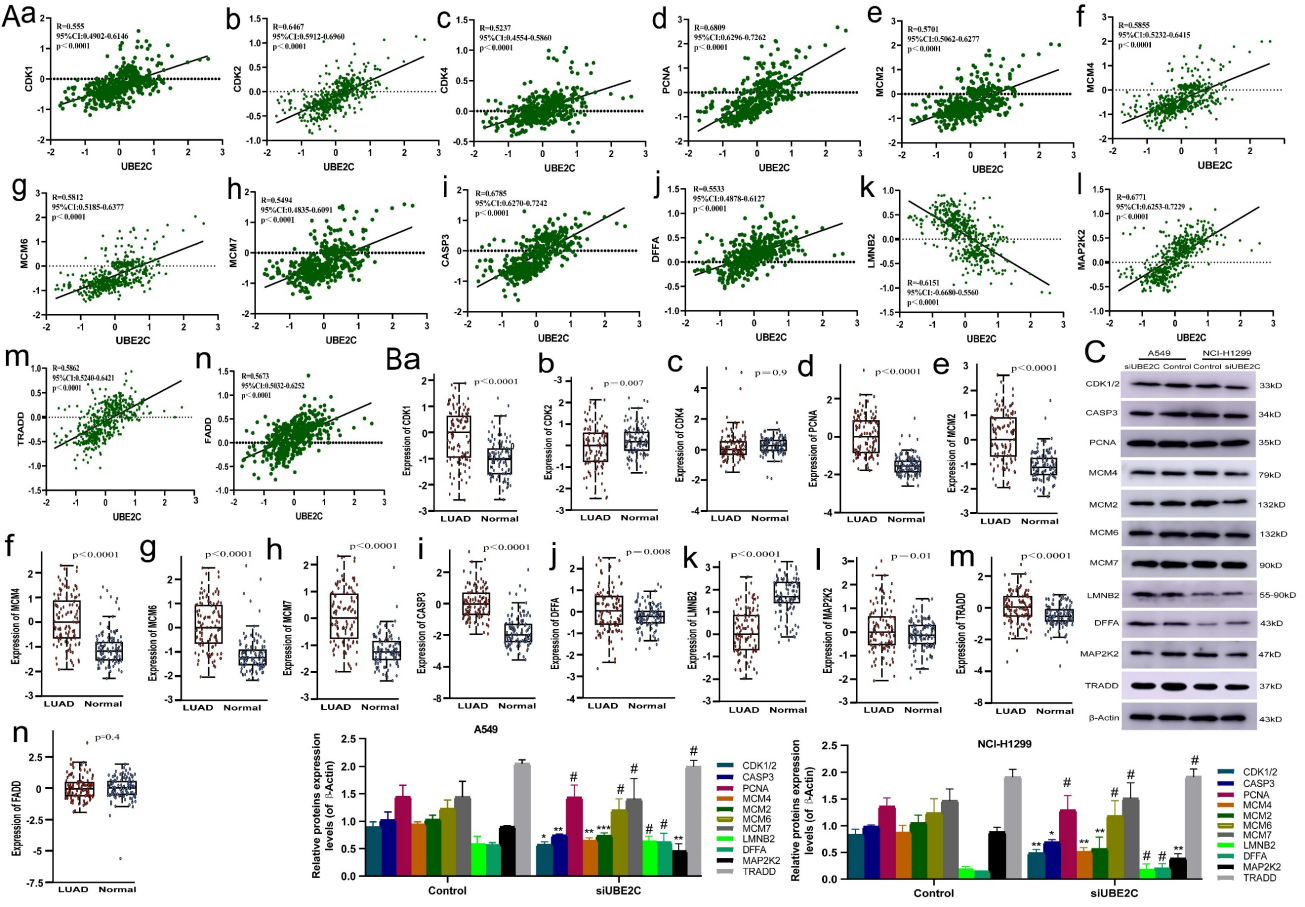
Correlation between cell cycle and apoptosis related genes and UBE2C in LUAD. (A) Correlation between mRNA expression of cell cycle and apoptosis related genes and UBE2C based on GEPIA2 database. (B) Differential protein expression of cell cycle and apoptosis-related genes in LUAD compared with normal lung tissue. (C) After silencing UBE2C, the expression levels of CDK1/2, CASP3, PCNA, MCM4, MCM2, MCM6, MCM7, LMNB2, DFFA, MAP2K2 and TRADD in both NCI-H1299 and A549 cells (^#^*p*>0.05, ^*^p < 0.05, ^**^p < 0.01, ^***^p < 0.001, ^****^p < 0.0001).

Subsequently, we explored the potential mechanism of overexpression of UBE2C in promoting cell cycle progression and suppressing apoptosis by analyzing the correlation between UBE2C and mRNA expression levels of genes related to human common cell cycle and apoptosis from GEPIA2 database (correlation coefficients ≥ 0.5 for UBE2C-related genes and ≥ 0.2 for apoptosis-related genes) (Supplementary Table 1). Then, protein expression data of human LUAD was downloaded from the CTAPAC database and those gene expressions were correlated with UBE2C from a proteomic perspective, resulting in a significant correlation of 14 genes with UBE2C (p<0.05, R≥5, Figure 6Aa-n). We based our analysis on protein expression data from CTPAC database for these 14 genes and found that CDK2 showed low expression in LUAD, which contrasts with the positive correlation between UBE2C and CDK4/CDK2 mRNA expression previously reported, and it needs to be removed from our current analysis. So, CDK4, FADD and CDK2 need to be removed from our analysis in this study. And we analyzed the expression levels of the remaining 11 genes (CDK1, CASP3, PCNA, MCM2, MCM4, MCM6, MCM7, LMNB2, DFFA, MAP2K2 and TRADD) after transfecting siRNA UBE2C into NCI-H1299 and A549 cells. We performed Western blot analysis to detect the total protein expression of these genes in the cells transfected with siRNA UBE2C. After silencing UBE2C, we observed a decrease in the expression levels of CDK1/2, CASP3, MCM4, MCM2, and MAP2K2 in both NCI-H1299 and A549 cells, while the expression levels of PCNA, MCM6, MCM7, LMNB2, DFFA, and TRADD remained unchanged (Figure 6C). Therefore, it can be inferred that UBE2C may regulate CDK1, MCM2, and MCM4 expression through certain mechanisms, thereby influencing the cell cycle progression of LUAD. Additionally, UBE2C may also regulate CASP3 and MAP2K2 expressions, further affecting the apoptosis of LUAD cells, thus promoting the progress of LUAD.

### Downregulation of UBE2C suppressed LUAD cell growth via inhibition of the Akt-mTOR signaling pathway in LUAD

Previous studies have found that UBE2C can promote the progression of NSCLC through autophagy, but the specific pathway mechanism remains unclear. The Akt-mTOR signaling pathway, an essential negative regulator of autophagy, is of great importance 5. We systematically evaluated UBE2C expression and somatic changes in Akt-mTOR signaling pathway using proteomics, and found that UBE2C expression was significantly positively correlated with changes in Akt-mTOR signaling pathway-altered [Figure 5F(a), p = 1.53e-11]. Then, we evaluated the expression levels of total and phosphorylated proteins of key molecules in the Akt-mTOR signaling pathway [Figure 5F(b)]. After 48-h treatment with si-UBE2C, phosphorylation levels of Akt and mTOR were significantly reduced in the si-UBE2C group compared with the control group, indicating that si-UBE2C has a chain reaction effect on the inactivation of the Akt-mTOR pathway. These findings suggest that UBE2C attenuation may be involved in autophagy by inhibiting the Akt-mTOR signaling pathway to affect LUAD cell proliferation, thereby inhibiting the progression of LUAD.

### UBE2C might be a potential immunotherapy target in LUAD

As mentioned above, we found that a positive correlation between UBE2C expression and tumor grade (Figure 2C), as well as stage (Figure 2D) in LUAD patients. This finding suggests that overexpressed UBE2C may contribute to the metastasis and invasion of LUAD. Previous research indicated that immune cell infiltration in tumor cells is closely associated with lymph node metastasis and prognosis in LUAD 22.

**Figure 7:**
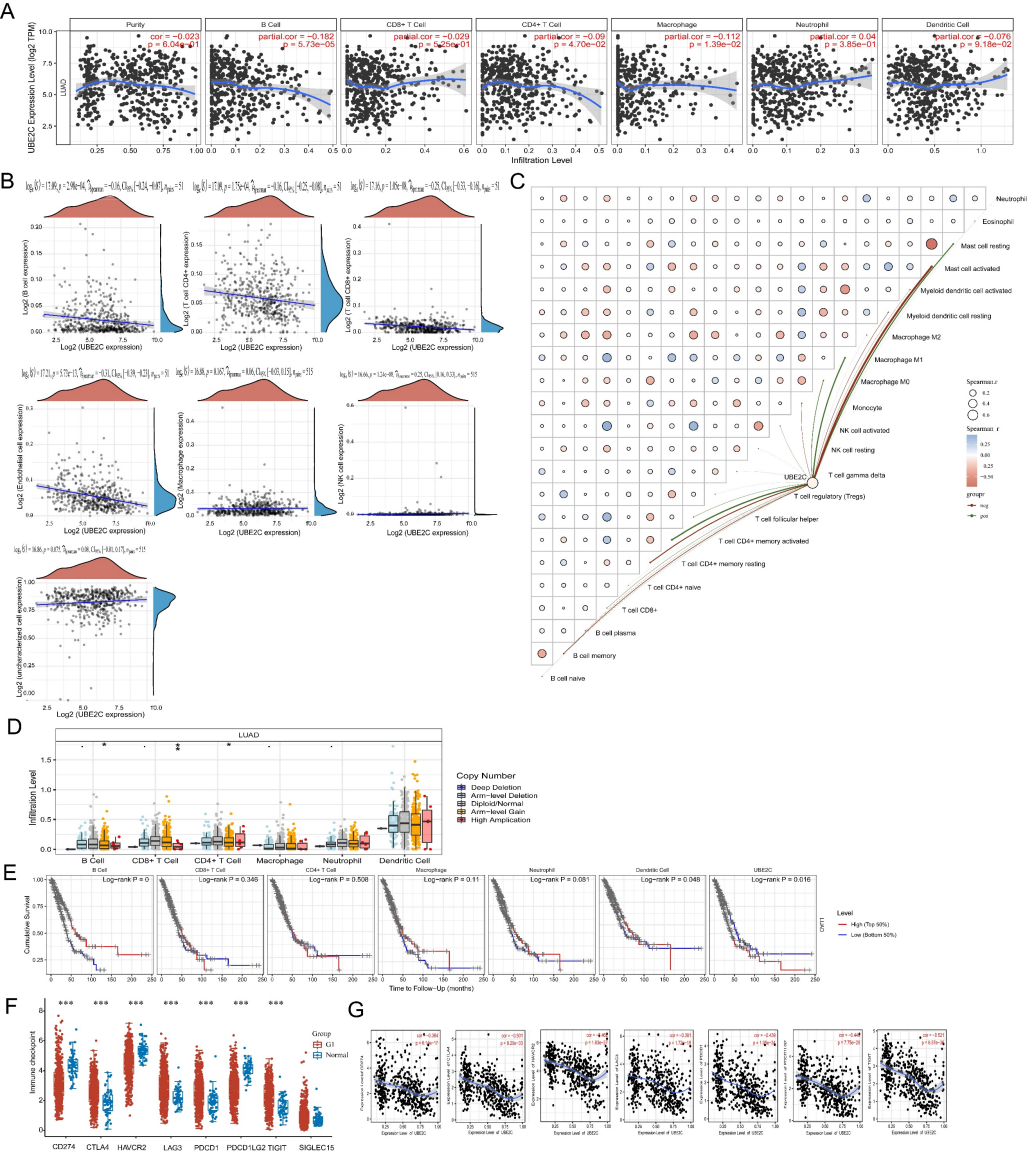
Correlation between the expression of UBE2C and immune infiltration. (A) Correlation between UBE2C expression and immune infiltrating cells in LUAD through TIMER database; (B-C) EPIC immune (B) and CIBERSORTC immune (C) correlations between UBE2C expression and immune score with Spearman. (D) Comparison of tumor infiltration levels among tumors with different somatic copy number alterations for UBE2C by SCNA module based on TIMER database. (E) Kaplan-Meier plots for immune infiltrates and UBE2C expression. (F) The heatmap of immune-checkpoint-related gene expression. (G) Correlation between UBE2C expression and CD274, CTLA4, HAVCR2, LAG3, PDCD1, PDCD1LG2, and TIGIT by TIMER database. (*p < 0.05, **p < 0.01, ***p < 0.001, ****p < 0.0001).

To analyze the correlation between UBE2C expression and immune infiltrating cells in LUAD, we utilized the TIMER database. And the correlation between the expression of UBE2C and specific immune infiltration cell markers in lung adenocarcinoma based on TIMER and GEPIA2 database in Supplementary Table 2. Our analysis revealed a negative correlation between UBE2C expression and the levels of B cells (r = -0.182, p = 5.73e-5), CD4+ T cells (r = -0.09, p = 4.70e-2), and macrophages (r = -0.112, p = 1.39e-2) (Figure 7A). Moreover, we conducted EPIC immune correlation analysis using Spearman to examine the association between UBE2C expression and immune score (Figure 7B). At the same time, CIBERSORT analysis revealed a correlation between the expression level of UBE2C and the infiltration of immune cells in tumors (Figure 7C). Furthermore, we observed a close association between UBE2C CNV and the degree of infiltration of B cells, CD4+ T cells, and CD8+ T cells (Figure 7D). These findings indicate that UBE2C expression may influence the infiltration of various immune cells in the immune microenvironment of LUAD, particularly B cells and CD4+ T cells. Previous studies have highlighted the importance of B cells, macrophages, CD4+ T cells, and dendritic cells as crucial immune cells with a wide range of anti-tumor effects 23-25. Hence, we speculate that the overexpression of UBE2C inhibits the invasion of B cells and CD4+ T cells, thus accelerating tumor progression in LUAD. Consequently, we believe that the high expression of UBE2C in LUAD patients may hinder the anti-tumor immune response, suggesting its significant role in the immune regulation of LUAD. UBE2C may also serve as a predictive biomarker for immune response. Additionally, we observed a significant correlation between dendritic cell infiltration (p = 0.048) and UBE2C expression (p = 0.016) with LUAD prognosis (Figure 7E). This indicates that patients in the low Dendritic Cell expression group have a worse prognosis compared to those in the high Dendritic Cell expression group (Figure 7E).

### Relationship between the expression of UBE2C and the therapeutic efficacy of tumor immune checkpoint in LUAD

In order to understand the relationship between the expression of UBE2C and the immune checkpoint gene in LUAD. We explored the predictive effect of UBE2C expression on the efficiency of immune checkpoint inhibitors in LUAD patients. Firstly, we analyzed the distribution of immune checkpoint genes' expressions in LUAD tissues and normal tissues. We discovered that expression levels of CD274, HAVCR2, and PDCD1LG2 in LUAD were significantly lower than those in normal lung tissue (Figure 7F; *p* < 0.001). On the other hand, the expression levels of CTLA4, LAG3, PDCD1, and TIGIT in LUAD were significantly higher than those in normal lung tissue **(**Figure 7F; *p* < 0.001). Unfortunately, there was no noticeable difference in SIGLEC15 expression between LUAD and normal lung tissue. Finally, we utilized the TIMER database to examine the correlation between UBE2C expression and those aforementioned genes. We observed a significant negative correlation between the expression of UBE2C and the expressions of CD274 (R = -0.364, p = 6.14e-17), CTLA4 (R = -0.501, p = 9.20e-33), HAVCR2 (R = -0.462, p = 1.83e-27), LAG3 (R = -0.381, p = 1.72e-18), PDCD1 (R = -0.439, p = 1.05e-24), PDCD1LG2 (R = -0.449, p = 7.75e-26), and TIGIT (R = -0.521, p = 8.57e-36) (Figure 7G).

### The expression of UBE2C and diagnosis value in pan-cancer

**Figure 8:**
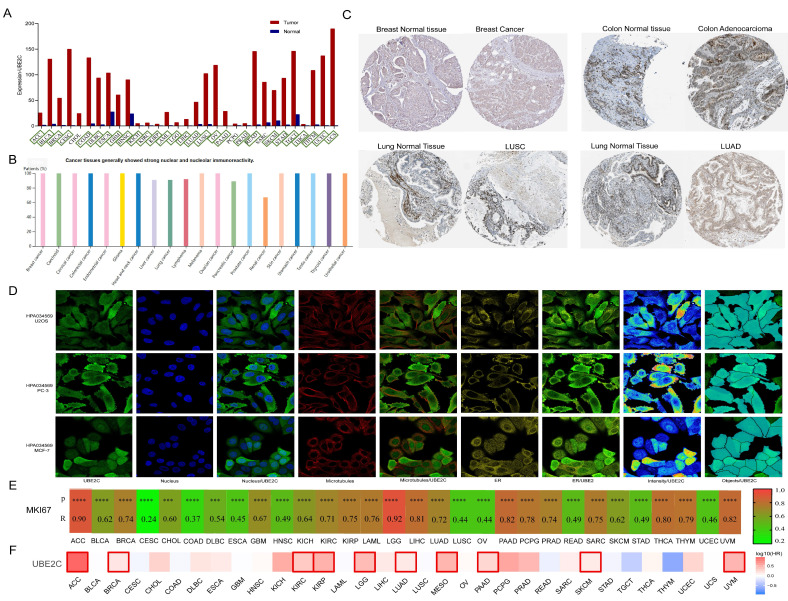
UBE2C expression and prognostic value in pan-cancer from different databases. (A) UBE2C expression in pan-cancer in GEPIA2. (B) UBE2C expression in pan-cancer from the HPA database. (C) Some typical immunohistochemical data. (D) Immunofluorescence staining of the subcellular localization of UBE2C in HPA database. (E) Pan-cancer correlation between UBE2C and MKi67. (F) The Kaplan-Meier curves for overall survival in pan-cancer (*, *p* < 0.05; **, *p* < 0.01; ***, *p* < 0.001, ns: no statistical differences).

The GEPIA2 database was utilized to analyze the expression of UBE2C in various cancer types and normal tissues. In comparison to matched normal tissues, UBE2C demonstrated significant overexpression in 28 different cancers, including ACC, BLCA, BRCA, CESC, COAD, DLBC, ESCA, GBM, HNSC, KICH, KIRC, KIRP, LAML, LGG, LIHC, LUAD, LUSC, OV, PAAD, PRAD, READ, SKCM, STAD, TGCT, THCA, THYM, UCEC, and UCS (Figure 8A; all* p*<0.05). According to data from the HPA database, UBE2C exhibited high expression levels in BRCA, Carcinoid, CESC, COAD, GBM, HNSC, LIHC, lung cancer, lymphoma, OV, PAAD, PRAD, KIRC, KIRP, KICH, SKCM, STAD, TGCT, and THCA (Figure 8B), and representative immunohistochemical results are displayed in Figure 8C. Subsequently, the subcellular distribution of UBE2C was determined via immunofluorescence localization in MCF-7, PC-3, and U2OS cells, revealing that UBE2C primarily localized to the nucleus, microtubules, and endoplasmic reticulum (ER) (Figure 8D).

Generally speaking, Ki67 (also known as MKI67, MIB-1, PPP1R105) is a very important biomarker for the proliferation index of cancers. It is used to determine the malignancy of tumors, evaluate the prognosis of patients, and determine whether the patient is sensitive to chemotherapy in some cancers 26. Correlation analysis was conducted between Ki67 and UBE2C in pan-cancer through the GEPIA2 database, and it was found that UBE2C was moderately to highly correlated with the Ki67 index in 33 tumors except for CESC and COAD (R > 0.4) (Figure 8E). The scatterplots for the correlation analysis of Ki67 and UBE2C expression in pan-cancer are shown in Supplementary Figure 2.

### Prognostic value of UBE2C in pan-cancer

Furthermore, the GEPIA2 database was utilized to examine the prognostic implications of UBE2C in various types of cancer. The analysis revealed that elevated UBE2C expression was associated with unfavorable OS in ACC, BRCA, KIRC, KIRP, LGG, LUAD, MESO, PAAD, SKCM, and UVM (Figure 8F, *p* < 0.05). These findings indicate that UBE2C serves as an independent prognostic marker for ACC, BRCA, KIRC, KIRP, LGG, LUAD, MESO, PAAD, SKCM, and UVM.

### Correlation between UBE2C and immune cell infiltration in pan-cancer

**Figure 9:**
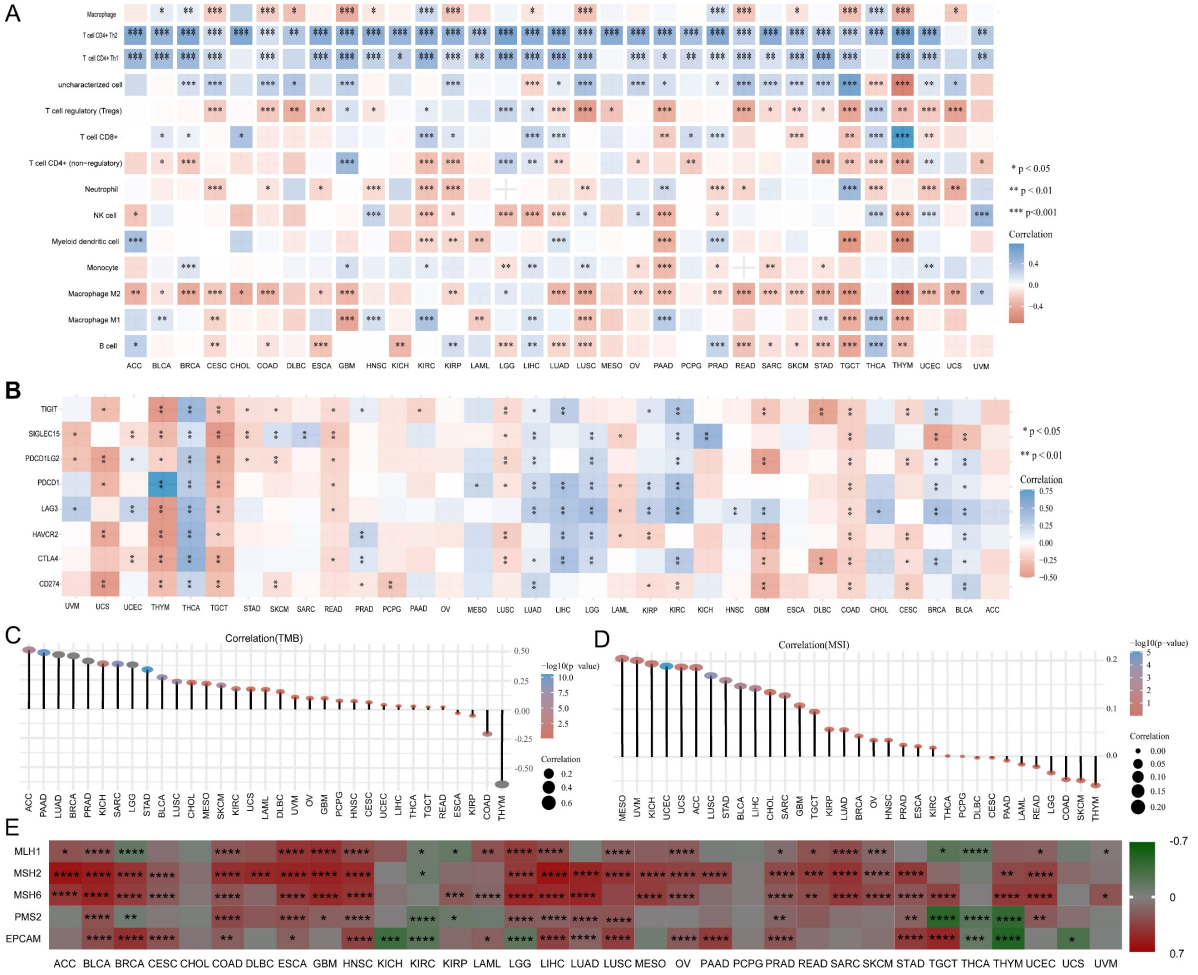
Correlation between UBE2C and immune. (A)Correlation between UBE2C and 14 immune cells in pan-cancer. (B) Correlation between UBE2C and immune checkpoints. (C-E) The correlation of UBE2C expression with TMB (C), MSI (D) and (E). TMB: tumor mutational burden; MSI: Microsatellite Instability; LUAD: Lung adenocarcinoma. (* p < 0.05, ** p < 0.01, *** p < 0.001, **** p < 0.0001).

We investigated the association between UBE2C expression and the infiltration of 14 different types of immune cells, using Spearman correlations. Our findings revealed that UBE2C was positively correlated with the levels of Th2 infiltration in 33 types of cancers, with the exception of UCS (Figure 9A). Additionally, UBE2C expression showed a positive correlation with Th1 infiltration in 28 types of cancers, and a negative correlation with macrophage M2 infiltration in 24 types of cancers, excluding LGG and UVM (Figure 9A). Furthermore, UBE2C exhibited significant positive or negative correlations with the infiltration of 12 other immune cells in the majority of the cancers.

Then, immune checkpoints analysis shown that UBE2C had a negative correlation with immune checkpoints, such as CD274(PD-1), PDCD1(PD-L1), CTLA4, TIGIT, LAG3, HAVCR2, and PDCD1LG2 in a great many cancers (Figure 9B). As we known, TMB (tumor mutational burden) and MSI (microsatellite instability) were significantly linked with immune checkpoint inhibitor (ICIs) sensitivity 27. And relationship between UBE2C and TMB and MSI were analyzed. We found that there was a significant positive correlation between UBE2C and TMB in 33 cancers except ESCA, KIRP, COAD and THYM, and there was a negative correlation between UBE2C and TMB in ESCA, KIRP, COAD and THYM (Figure 8C). Moreover, UBE2C displayed a positive association with MSI in MESO, UVM, KICH, UCEC, UCS, ACC, LUSC, STAD, BLCA, LIHC, CHOL, SARC, GBM, TGCT, KIRP, LUAD, BRCA, OV, HNSC, PRAD, ESCA, KIRC and a negative association in THYM, SKCM, COAD, LGG, READ, LAML, PAAD and CESC (Figure 8D). What's more, UBE2C expression was positively linked with MLH1, MSH2, MSH6 and PMS2 in most cancers except KIRC and THCA, but was negatively correlated with MLH1 and PMS2 in BRCA, KIRP, TGCT and THYM (Figure 9E,* p* < 0.05&*p* < 0.001&* p* < 0.0001).

### Single-Cell functional analysis of UBE2C in pancancer

**Figure 10:**
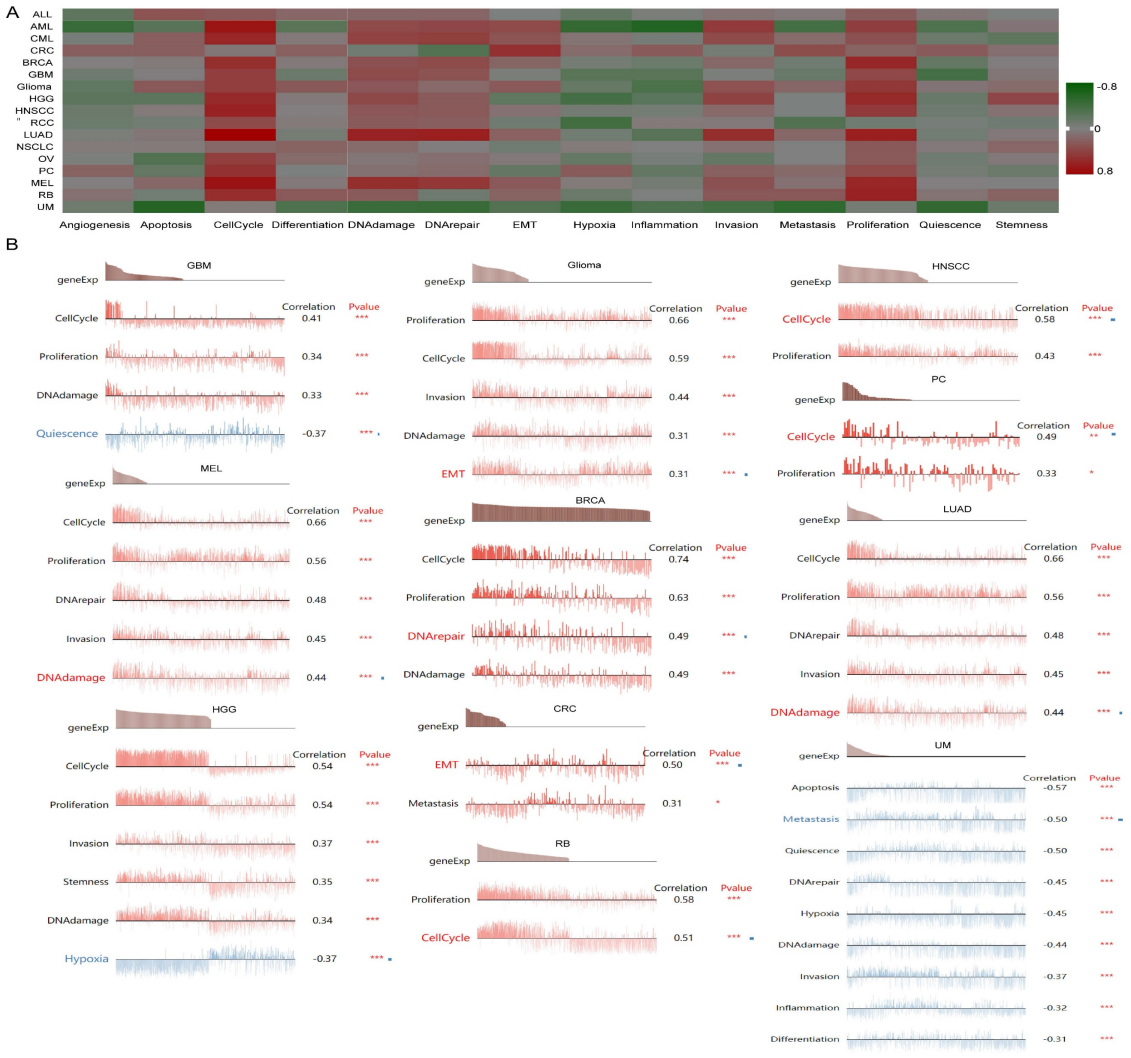
The function of UBE2C in single-cell functional analysis from the CancerSEA database. (A) Functional status of UBE2C in different human cancers. (B) Correlation analysis between functional status and UBE2C in GBM, glioma, HNSCC, MEL, BRCA, LUAD, PC, HGG, CRC, RB and UM (* p < 0.05, ** p < 0.01, *** p < 0.001).

To further understand the latent role of UBE2C in tumors, we investigated the function of UBE2C at the single-cell level using CancerSEA (Figure 10A). The findings revealed that UBE2C was positively associated with cell cycles of AML, CML, BRCA, GBM, glioma, HGG, HNSCC, LUAD, RCC, OV, PC, MEL, and RB. Additionally, UBE2C had a positive relationship with both cell cycle and proliferation in in AML, CML, BRCA, GBM, Glioma, HGG, HNSCC, LUAD, MEL and RB (Figure 10A).

In GBM, UBE2C exhibited a positive correlation with cell cycle, DNA damage, and proliferation. Conversely, it showed a negative correlation with quiescence. UBE2C expression was found to be positively correlated with cell cycle, proliferation, DNA damage, invasion, and MET in Glioma. Similarly, in HNSCC and PC, UBE2C was positively associated with cell cycle and proliferation. In the case of MEL and LUAD, UBE2C expression was positively linked to cell cycle, proliferation, DNA damage, invasion, and DNA repair (Figure 10B).

## Discussion

The role of UBE2C is to promote proliferation and inhibit autophagy in lung cancer cells 3. However, the detailed mechanism of UBE2C in promoting cancer progression, as well as its specific diagnostic, prognostic, and immunotherapeutic value in LUAD, have not been extensively studied. Although previous studies have analyzed the role of UBE2C in pan-cancer 28, there is a lack of further research on the mechanism of UBE2C in pan-cancer and its prognostic and immunotherapeutic value. In our study, we demonstrated that downregulating UBE2C expression inhibited the proliferation of LUAD cells by inducing G2/M phase arrest, inhibiting cell apoptosis, and suppressing the Akt-mTOR signaling pathway.

The cyclin-dependent kinase (CDK) family includes 20 different types of enzymes (CDK1-20), which have been proven to play a crucial role in various types of cancer, including melanoma and lung cancer, as oncogenes. CDK1 is unique among these CDKs as it plays a pivotal role in cell cycle progression by promoting G2/M phase transition and G1/S phase transition, and also G1 progression. Inhibition of CDK1 expression and activation effectively suppresses the function of many cancer cells 29. The six conserved proteins (MCM2, MCM3, MCM4, MCM5, MCM6 and MCM7) form a hexameric complex as a DNA helicase, playing a crucial role in the initiation of DNA replication. MCM2 is a core subunit of the essential eukaryotic DNA helicase, which plays a vital role in DNA replication and is considered a potential biomarker for cancer diagnosis, prognosis, and chemotherapy sensitivity 30. MCM4 is highly expressed in LUAD tumors and cells, and its overexpression promotes LUAD cell proliferation while suppressing apoptosis and affecting cell cycle 31. Caspases are a family of 15 members that play an important role in programmed cell death. Among them, caspase 3 is a typical apoptosis-related gene. When it is activated by caspase 8 or caspase 9, it regulates many other key functional proteins inside cells, leading to cell apoptosis. Many cancer therapies, including chemotherapy drugs, radiotherapy, or immunotherapy, can trigger tumor cell apoptosis through the activation of caspase 3 32. The role of the ERK signaling pathway in cancer is considered to be the most prominent among tumors, MEK2 mutations promote the activation of ERK1 and ERK2, leading to increased cell proliferation and survival. Recently approved or ongoing clinical trials of drugs that inhibit MEK2 33. In our study, it is believed that UBE2C may regulate CDK1, MCM2, and MCM4 expression through certain mechanisms, thereby affecting the cell cycle of LUAD, promoting its progression. Furthermore, it is believed that UBE2C may regulate CASP3 and MAP2K2 through certain mechanisms, thus influencing the apoptosis of LUAD, promoting its progress.

Previous have indicated that both TMB and MSI serve as reliable biomarkers for prognosing various cancers and predicting the efficacy of immunotherapy for multiple tumor types. Specifically, tumors with high TMB and MSI-high exhibit better response rates to immunotherapy 34, 35. Our research revealed a positive correlation between UBE2C expression and immune scores within the TME matrix across four cancer types (THCA, LUAD, LIHC, and LGG), while a negative correlation was observed in the case of seven cancer types (UCS, TGCT, READ, LUSC, LAML, and COAD) (Figure 9A). These findings suggest a close association between UBE2C expression and immune cell infiltration in tumors, ultimately affecting the prognosis of cancer patients. This discovery opens up new opportunities for developing immunosuppressive agents and innovative treatment strategies. Notably, immune checkpoint genes play a crucial role in the therapeutic application of immune checkpoint inhibitors (ICI) against cancer and are considered effective immunotherapeutic targets 36. We also identified a positive correlation between UBE2C expression and common immune checkpoint markers such as PD-L1, PD-1, CTLA4, TIGIT, HAVCR2, PDCD1LG2, and LAG3 (Figure 9B). These findings further suggest that UBE2C may serve as a novel target for tumor immunotherapy. Our study highlights the potential of UBE2C as a predictive marker for immunotherapy efficacy and underscores its significance as a potential target for tumor immunotherapy. Our findings provide crucial insights into the potential mechanisms of UBE2C in the development of various types of tumors. However, this is just the tip of the iceberg, and there's still much to explore in the field of UBE2C and its potential role in tumorigenesis.

## Conclusions

Our research confirmed that UBE2C could serve as a biomarker for the diagnosis, treatment and prognosis of pan-cancers including LUAD.

## Supplementary Material

Supplementary figures and tables.

## Figures and Tables

**Table 1:** Multivariate cox regression analysis of OS for UBE2C expression.

Overall Survival	HR	95%CI	*p*-value
UBE2C: low (1798) vs high (1632)	1.69	1.5-1.91	< 1e-16
Gender: Female (925) vs Male (1545)	1.77	1.48-2.13	3.60e-10
Histology: LUAD (1308) vs LUSC (931)	1.62	1.43-1.83	2.00e-14
Stage: Stage1 (907) vs Stage2 (478) vs Stage3(112) vs Stage4 (4)	2.61	1.92-3.55	1.90e-10
Grade: GradeI (189) vs GradeII (302) vs GradeIII (75)	1.48	1.14-1.92	0.003
AJCC StageT: T1(467) vs T2(672) vs T3(100) vs T4(44)	1.54	1.24-1.91	7.80e-05
AJCC StageN: N0 (848) vs N1(296) vs N2(105)	1.53	1.24-1.9	9.10e-05
Smoking history: exclude those never smoked (1007) vs Only those never smoked (247)	2.11	1.65-2.71	1.90e-09

## Abbreviations

ACC: adrenocortical carcinoma
BLCA: bladder urothelial carcinoma
BRCA: breast invasive carcinoma
CESC: cervical squamous cell carcinoma and endocervical adenocarcinoma
CHOL: cholangiocarcinoma
COAD: colon adenocarcinoma
DLBC: lymphoid neoplasm diffuse Large B-cell lymphoma
ESCA: esophageal carcinoma
GBM: glioblastoma multiforme
HNSC: head and neck squamous cell carcinoma
KICH: kidney chromophobe
KIRC: kidney renal clear cell carcinoma
KIRP: kidney renal papillary cell carcinoma
LAML: acute myeloid leukemia
LGG: brain lower grade glioma
LIHC: liver hepatocellular carcinoma
LUAD: lung adenocarcinoma
LUSC: lung squamous cell carcinoma
MESO: mesothelioma
OV: ovarian serous cystadenocarcinoma
PAAD: pancreatic adenocarcinoma
PCPG: pheochromocytoma and paraganglioma
PRAD: prostate adenocarcinoma
READ: rectum adenocarcinoma
SARC: sarcoma
SKCM: skin cutaneous melanoma
STAD: stomach adenocarcinoma
TGCT: testicular germ cell tumors
THCA: thyroid carcinoma
THYM: thymoma
UCEC: uterine corpus endometrial carcinoma
UCS: uterine carcinosarcoma
UVM: uveal melanoma
