# Supplementary material for: UBE2C is a diagnosis and therapeutic biomarker involved in immune infiltration of cancers including lung adenocarcinoma
Source: J Cancer. 2024 Jan 27;15(6):1701–17. doi: 10.7150/jca.92473 (PMC10869987; doi:10.7150/jca.92473)
Supplement: Supplementary file 1 — Supplementary figures and tables. [file jcav15p1701s1.pdf]

Supplementary Figure1 Receiver operating characteristic (ROC) curve analysis between LUAD patients and healthy control of the expression of UBE2C from GEO databases.

Supplementary Figure2 The scatter plots for correlation analysis of Ki67 and UBE2C expression in pan-cancer.

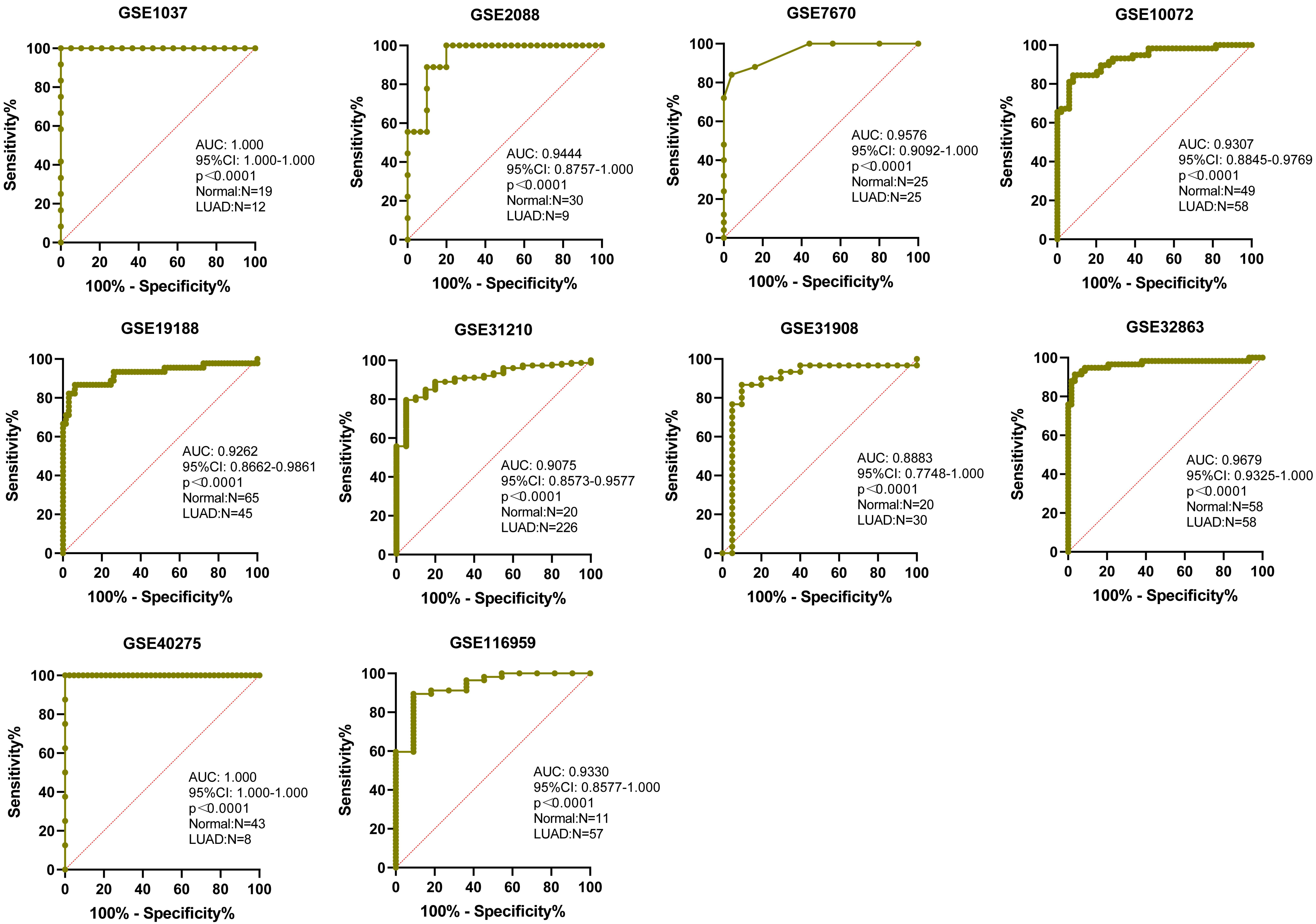

GSE1037
Sensitivity%
100% - Specificity%
AUC: 1.000
95%CI: 1.000-1.000
p<0.0001
Normal:N=19
LUAD:N=12
GSE2088
Sensitivity%
100% - Specificity%
AUC: 0.9444
95%CI: 0.8757-1.000
p<0.0001
Normal:N=30
LUAD:N=9
GSE7670
Sensitivity%
100% - Specificity%
AUC: 0.9576
95%CI: 0.9092-1.000
p<0.0001
Normal:N=25
LUAD:N=25
GSE10072
Sensitivity%
100% - Specificity%
AUC: 0.9307
95%CI: 0.8845-0.9769
p<0.0001
Normal:N=49
LUAD:N=58
GSE19188
Sensitivity%
100% - Specificity%
AUC: 0.9262
95%CI: 0.8662-0.9861
p<0.0001
Normal:N=65
LUAD:N=45
GSE31210
Sensitivity%
100% - Specificity%
AUC: 0.9075
95%CI: 0.8573-0.9577
p<0.0001
Normal:N=20
LUAD:N=226
GSE31908
Sensitivity%
100% - Specificity%
AUC: 0.8883
95%CI: 0.7748-1.000
p<0.0001
Normal:N=20
LUAD:N=30
GSE32863
Sensitivity%
100% - Specificity%
AUC: 0.9679
95%CI: 0.9325-1.000
p<0.0001
Normal:N=58
LUAD:N=58
GSE40275
Sensitivity%
100% - Specificity%
AUC: 1.000
95%CI: 1.000-1.000
p<0.0001
Normal:N=43
LUAD:N=8
GSE116959
Sensitivity%
100% - Specificity%
AUC: 0.9330
95%CI: 0.8577-1.000
p<0.0001
Normal:N=11
LUAD:N=57

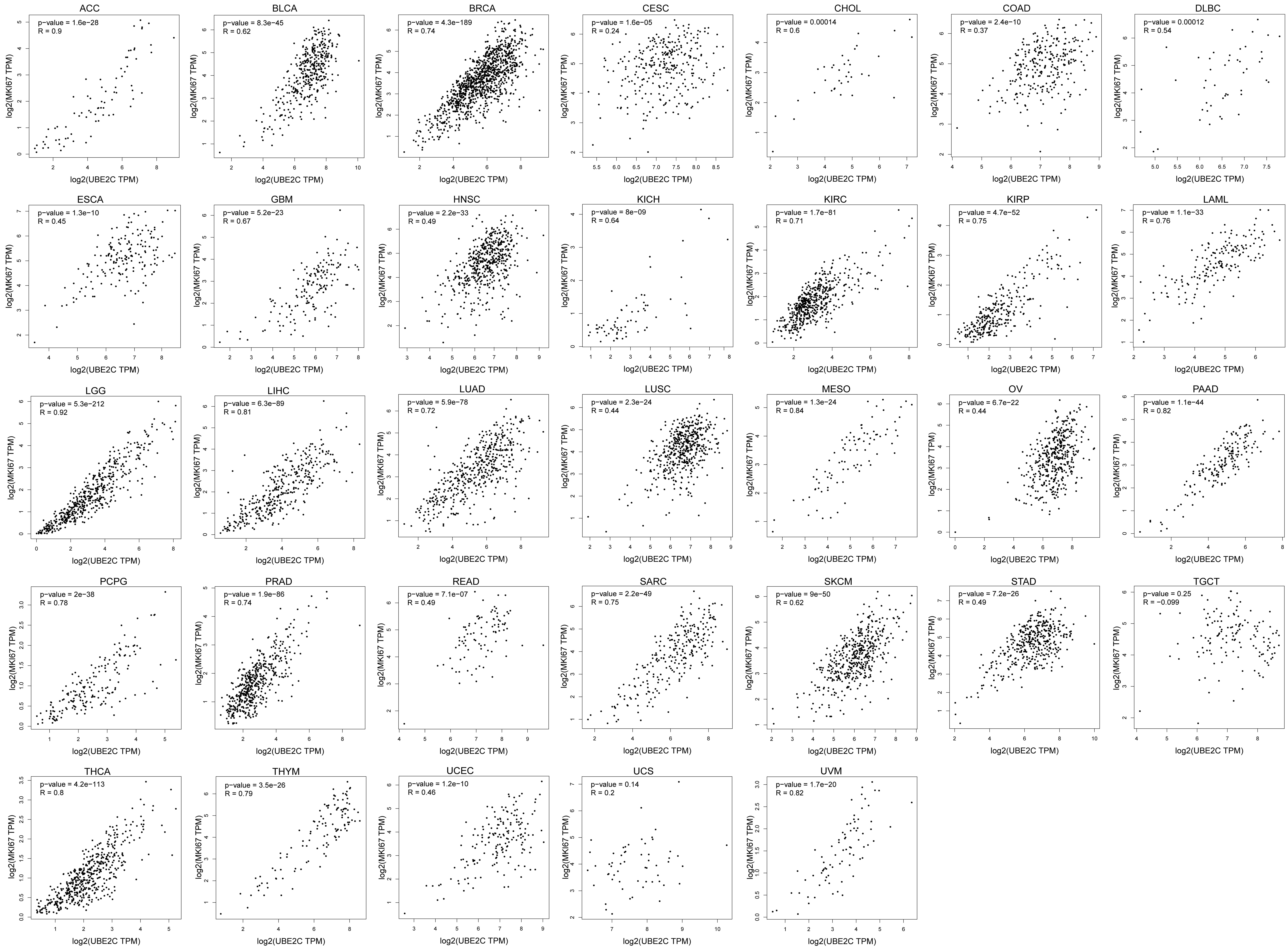

ACC
p-value = 1.6e-28
R = 0.9
BLCA
p-value = 8.3e-45
R = 0.62
BRCA
p-value = 4.3e-189
R = 0.74
CESC
p-value = 1.6e-05
R = 0.24
CHOL
p-value = 0.00014
R = 0.6
COAD
p-value = 2.4e-10
R = 0.37
DLBC
p-value = 0.00012
R = 0.54
ESCA
p-value = 1.3e-10
R = 0.45
GBM
p-value = 5.2e-23
R = 0.67
HNSC
p-value = 2.2e-33
R = 0.49
KICH
p-value = 8e-09
R = 0.64
KIRC
p-value = 1.7e-81
R = 0.71
KIRP
p-value = 4.7e-52
R = 0.75
LAML
p-value = 1.1e-33
R = 0.76
LGG
p-value = 5.3e-212
R = 0.92
LIHC
p-value = 6.3e-89
R = 0.81
LUAD
p-value = 5.9e-78
R = 0.72
LUSC
p-value = 2.3e-24
R = 0.44
MESO
p-value = 1.3e-24
R = 0.84
OV
p-value = 6.7e-22
R = 0.44
PAAD
p-value = 1.1e-44
R = 0.82
PCPG
p-value = 2e-38
R = 0.78
PRAD
p-value = 1.9e-86
R = 0.74
READ
p-value = 7.1e-07
R = 0.49
SARC
p-value = 2.2e-49
R = 0.75
SKCM
p-value = 9e-50
R = 0.62
STAD
p-value = 7.2e-26
R = 0.49
TGCT
p-value = 0.25
R = -0.099
THCA
p-value = 4.2e-113
R = 0.8
THYM
p-value = 3.5e-26
R = 0.79
UCEC
p-value = 1.2e-10
R = 0.46
UCS
p-value = 0.14
R = 0.2
UVM
p-value = 1.7e-20
R = 0.82
log2(MKI67 TPM)
log2(UBE2C TPM)

Supplementary Table 1 The mRNA expression of cell cycle and apoptosis related genes correlated with UBE2C in LUAD

Cell cycle-related genes			Apoptosis-related genes		
Gene	R	*p*	Gene	R	*p*
CDC45	0.53	0	ACTG1	0.32	1.00E-12
_CDT1	0.5	0	BIRC5	0.82	8.30E-120
DBF4	0.59	0	CASP2	0.34	7.00E-15
DBF4B	0.5	0	CASP3	0.25	2.00E-08
MCM2	0.53	0	DFFA	-0.37	3.00E-17
MCM4	0.53	0	FOS	-0.38	1.40E-17
MCM6	0.54	0	GZMB	0.41	2.80E-21
MCM7	0.54	0	ITPR1	-0.33	9.00E-14
MTBP	0.54	0	LMNB1	0.66	1.40E-62
ORC6	0.58	0	LMNB2	0.63	8.40E-56
RBL1	0.5	0	MAP2K2	0.25	4.10E-08
CCNB1	0.64	0	MAPK8	0.24	1.70E-07
CCNB2	0.6	0	PARP1	0.26	4.00E-09
AURKB	0.86	9.30E-142	PARP2	0.44	2.60E-24
CDCA5	0.82	1.10E-118	PTPN13	-0.36	8.40E-16
ESCO2	0.61	8.00E-50	TRADD	-0.24	5.90E-08
SGO1	0.81	3.30E-111	PMAIP1	0.4	2.10E-20
CCNA2	0.81	3.50E-111	DFFA	0.3	1.70E-11
CDC20	0.86	6.50E-145	ACTB	0.3	7.90E-12
CDC25A	0.75	5.40E-87	ERN1	-0.22	6.20E-07
CDC25C	0.62	0.00E+00	FADD	0.23	4.50E-07
CDK1	0.8	1.80E-109	GADD45G	-0.2	1.40E-05
CDK2	0.57	7.90E-44	ITPR2	0.21	2.60E-06
CDK4	0.53	3.00E-36	PARP3	-0.24	8.30E-08
CHEK1	0.75	6.90E-89	PARP4	-0.24	6.10E-08
CHEK2	0.59	8.30E-46	PDPK1	-0.22	1.60E-06
FBXO5	0.7	1.10E-71	MAPK10	-0.45	2.40E-25
PCNA	0.59	9.50E-47			
PKMYT1	0.73	4.90E-80			
PLK1	0.79	1.30E-103			
PRKDC	0.5	1.40E-31			
SKP2	0.6	4.50E-48			
ESPL1	0.58	0			
CDK1	0.53	0			

Supplementary Table 2 Correlation between the expression of UBE2C and specific immune infiltration cell markers in lung adenocarcinoma based on TIMER and GEPIA2 database.

Description	Gene markers	TIMER		GEPIA2	
		R	p	R	p
CD8+ T cell	CD8A	0.162	3.20E-04	0.16	0.00031
	IL2RA	0.195	1.27E-05	0.15	0.00072
	CD8B	0.168	1.83E-04	0.14	0.0019
T cell (general)	CD3D	0.054	2.32E-01	0.034	0.45
	CD3E	0.024	5.96E-01	-0.045	0.32
	CD2	0.026	5.66E-01	-0.044	0.33
B cell	CD19	0.065	1.47E-01	-0.066	0.15
	MS4A1	0.189	2.30E-05	-0.18	4.60E-05
	CD79A	0.075	9.44E-02	-0.11	0.015
Monocyte	CD86	0.06	1.80E-01	0.027	0.55
	CD14	0.101	2.43E-02	0.066	0.14
	CD33	0.167	1.91E-04	-0.16	0.00052
	CD115 (CSF1R)	0.072	1.13E-01	-0.085	0.061
TAM	CCL2	0.086	5.62E-02	0.067	0.14
	CD68	0.016	7.16E-01	-0.045	0.33
	IL10	0.011	8.13E-01	0.00051	0.99
M0 Macrophage	CD206(MRC1)	0.229	2.85E-07	-0.24	1.50E-07
	EFEMP2	0.047	3.02E-01	-0.013	0.77
	CD163	0.032	4.77E-01	0.08	0.078
M1 Macrophage	INOS (NOS2)	0.037	4.17E-01	0.068	0.13
	IRF5	0.079	7.84E-02	0.073	0.11
	COX2(PTGS2)	0.011	8.05E-01	0.027	0.56
M2 Macrophage	VSIG4	0.045	3.14E-01	-0.058	0.2
	MRC1	0.229	2.85E-07	-0.24	1.50E-07
	CD209	0.073	1.04E-01	0.04	0.38
	MS4A4A	0.068	1.32E-01	-0.091	0.045
	CD66b (CEACAM8)	0.369	2.57E-17	-0.39	3.80E-19
Neutrophils	CD11b (ITGAM)	0.079	7.87E-02	-0.12	0.0087
	B3GAT1	0.249	2.19E-08	-0.23	3.80E-07
	KIR3DL1	0.023	6.04E-01	0.13	0.0043
	CD7	0.284	1.28E-10	0.24	1.20E-07
	CCR7	0.195	1.29E-05	-0.18	5.80E-05
	KIR2DL1	0.084	6.20E-02	0.073	1.10E-01
Natural killer cell	KIR2DL3	0.188	2.53E-05	0.16	3.30E-04
	KIR2DL4	0.418	3.18E-22	0.39	3.30E-19
	KIR3DL1	0.105	1.98E-02	0.13	4.30E-03
	KIR3DL2	0.169	1.56E-04	0.11	1.80E-02
	KIR3DL3	0.203	5.48E-06	0.19	3.40E-05
	B3GAT1	0.249	2.19E-08	-0.23	3.80E-07

	CD7	0.284	1.28E-10	0.24	1.20E-07
	KIR2DS4	0.118	8.58E-03	0.11	1.20E-02
	HLA-DPB1	-0.349	1.37E-15	-0.32	7.00E-13
Dendritic cell	HLA-DQB1	-0.235	1.38E-07	-0.18	7.40E-05
	HLA-DRA	-0.281	2.17E-10	-0.27	8.10E-10
	HLA-DPA1	-0.292	3.53E-11	-0.28	4.60E-10
	BDCA-1(CD1C)	-0.462	2.16E-27	-0.44	7.50E-24
	BDCA-4(NRP1)	0.012	7.89E-01	-0.0077	8.70E-01
	THBD	-0.281	2.09E-10	-0.28	4.20E-10
	CD11c (ITGAX)	0.01	8.24E-01	-0.0034	9.40E-01
	T-bet (TBX21)	0.066	1.45E-01	0.036	4.40E-01
Th1	STAT4	-0.063	1.65E-01	-0.05	2.70E-01
	STAT1	0.355	4.54E-16	0.33	1.70E-13
	IFN-γ (IFNG)	0.332	3.49E-14	0.29	1.10E-10
	CCR5	0.032	4.80E-01	-0.018	7.00E-01
	CCR1	0.039	3.93E-01	0.017	7.10E-01
	IL12RB1	0.05	2.71E-01	0.016	7.30E-01
	TNF-α (TNF)	0.035	4.33E-01	0.022	6.30E-01
	GATA3	0.033	4.59E-01	0.025	5.90E-01
Th2	STAT6	-0.308	2.86E-12	-0.26	1.10E-08
	STAT5A	-0.075	9.46E-02	-0.086	5.80E-02
	CCR8	0.008	8.59E-01	-0.016	7.20E-01
	HAVCR1	-0.093	3.88E-02	-0.12	1.00E-02
	CCR4	-0.24	6.98E-08	-0.25	3.50E-08
	IL13	-0.005	9.09E-01	-0.066	1.50E-01
	CXCR5	-0.106	1.89E-02	0.0026	9.60E-01
Tfh	ICOS	0.061	1.75E-01	0.033	4.70E-01
	BCL6	-0.129	4.20E-03	-0.12	9.60E-03
	CXCR3	0.089	4.81E-02	0.059	1.90E-01
	IL21	0.183	4.34E-05	0.16	4.60E-04
	STAT3	-0.201	6.76E-06	-0.19	2.30E-05
Th17	IL21R	0.102	2.37E-02	0.058	2.00E-01
	IL23R	-0.176	8.88E-05	-0.19	2.20E-05
	CCR6	-0.398	3.62E-20	-0.37	3.90E-17
	IL17A	0.117	9.54E-03	0.11	1.20E-02
	FOXP3	0.071	1.14E-01	0.063	1.70E-01
Treg	NT5E	-0.091	4.40E-02	-0.089	4.90E-02
	IL7R	-0.161	3.42E-04	-0.15	1.40E-03
	CCR8	0.008	8.59E-01	-0.016	7.20E-01
	STAT5B	-0.101	2.45E-02	-0.11	1.50E-02
	TGFβ (TGFB1)	-0.072	1.12E-01	-0.039	3.90E-01
	PD-1 (PDCD1)	0.247	2.89E-08	0.2	1.60E-05
T cell exhaustion	CTLA4	0.143	1.41E-03	0.093	4.10E-02
	LAG3	0.309	2.16E-12	0.28	6.50E-10

	TIM-3 (HAVCR2)	0.046	3.06E-01	0.027	5.60E-01
	GZMB	0.472	9.88E-29	0.41	2.80E-21
	PDL1(CD274)	0.234	1.55E-07	0.22	7.50E-07
